# Supplementary material for: Self-supervised learning reveals clinically relevant histomorphological patterns for therapeutic strategies in colon cancer
Source: Nat Commun. 2025 Mar 8;16:2328. doi: 10.1038/s41467-025-57541-y (PMC11890774; doi:10.1038/s41467-025-57541-y)
Supplement: Supplementary file 1 — Supplementary Information [file 41467_2025_57541_MOESM1_ESM.pdf]

Supplementary Information

Self-Supervised Learning Reveals Clinically Relevant Histomorphological Patterns for Therapeutic Strategies in Colon Cancer

Bojing Liu[1,2,+], Meaghan Polack[3,+], Nicolas Coudray[2,4,#], Adalberto Claudio Quiros[5,#], Theodore Sakellaropoulos[2], Hortense Le[2], Afreen Karimkhan[6], Augustinus S.L.P. Crobach[7], J. Han J.M. van Krieken[8], Ke Yuan[5,9], Rob A.E.M. Tollenaar[3], Wilma E. Mesker[3], Aristotelis Tsirigos[2,6,*]

[1]Department of Medical Epidemiology and Biostatistics, Karolinska Institutet, Sweden.

[2]Applied Bioinformatics Laboratories, New York University Grossman School of Medicine, New York, New York, USA.

[3]Department of Surgery, Leiden University Medical Center, Leiden, The Netherlands.

[4]Department of Cell Biology, New York University Grossman School of Medicine, New York, New York, USA.

[5]Department of Computing Science, University of Glasgow, Glasgow, United Kingdom.

[6]Department of Pathology, New York University Grossman School of Medicine, New York, New York, USA.

[7]Department of Pathology, Leiden University Medical Center, Leiden, The Netherlands.

[8]Department of Pathology, Radboud University Medical Center, Nijmegen, The Netherlands.

[9]School of Cancer Sciences, University of Glasgow, Glasgow, Scotland, UK.

[+,#]Authors with equal contribution.

[*]Corresponding author: Aristotelis Tsirigos, Aristotelis.Tsirigos@nyulangone.org.

Supplementary Table 1: Tissue composition for all 47 HPCs

HPC	Super-cluster	Label	General description	Tumor epithelium	Tumor stroma	Immune cells	Other	HPC tissue type composition
0	Tumor stroma	Disorganized stroma	Mostly disorganized stroma, sometimes some aligned strands and sometimes some muscle tissue, immune cells in between, with a few isolated tumor cells	A couple isolated tumor cells	Primarily tumor stroma on tiles. Stroma-high tiles, more disorganized than aligned stroma	Some immune cells in hotspots, immune-high overall	Some muscle tissue	Stroma 70%, Muscle tissue 20%, Immune cells 10%
1	Tumor stroma	Edematous stroma	Loose, edematous and disorganized stroma with background, sometimes vessels and an artefact. Most likely not tumor-induced stroma but healthy serosa as there is also some fatty tissue	A couple isolated tumor cells	Primarily loose, edematous and disorganized stroma-high tiles with background, sometimes vessels with some erythrocytes. Most likely not tumor-induced stroma but healthy serosa looking at pattern, and there is also some fatty tissue	A couple immune cells, immune-low	Some fatty tissue	Stroma 80%, Fatty tissue 10%, Background 10%
2	Tumor stroma	Aligned stroma - well differentiated tumor	Mostly up until 50% stroma in aligned, straight strands. Well differentiated tumor in between, with a little dirty necrosis	Mostly well differentiated tumor epithelial cells	Mostly aligned, straight strands of tumor stroma on the stroma-low tiles	A couple immune cells, and some (dirty) necrosis, immune-low	N/A	Stroma 40%, Tumor epithelial cells 60%
3	Tumor epithelium	Moderately differentiated tumor	Well-moderately differentiated tumor, some high-grade dysplasia, and little stroma, (dirty) necrosis and background	Much well-moderately differentiated tumor epithelium	Little tumor stroma, stroma-low tiles	A couple immune cells, and some (dirty) necrosis, immune-low	Some high-grade dysplasia, some background. A couple vessels with some erythrocytes	Tumor epithelial cells 70%, Stroma 10%, Necrosis 10%, Background 10%
4	Tumor epithelium	Moderately differentiated tumor - aligned stroma	Well-moderately differentiated tumor, with in between strands of stroma, some immune cells and a few spots of dirty necrosis	Well-moderately differentiated tumor	Some strands of aligned tumor stroma, stroma-low tiles	Some immune cells, and a few spots of dirty necrosis, immune-high overall	N/A	Tumor epithelial cells 60%, Stroma 30%, Immune cells 10%
5	Necrosis	Necrotic	Mostly (dirty) necrosis, some tumor epithelial cells and some vessels, with background	A couple isolated tumor cells	Some aligned tumor stroma strands, stroma-low tiles	Primarily tiles containing (dirty) necrosis	Some vessels with dirty necrosis and some background or similarly fatty tissue or mucin	Necrosis 80%, Tumor epithelial cells 10%, Background 10%
6	Healthy and dysplastic colon tissue	Inflamed healthy colon tissue	Healthy and some dysplastic colon tissue, with many immune cells in proliferative and often vascularized stroma, aspect of inflammation	No tumor epithelium	No tumor stroma, only inflamed and vascularized 'healthy' stroma	Many immune cells	Primarily healthy and some low-grade dysplastic colon tissue with vascularized and proliferative aspect of stroma in between, some inflammation	Healthy or dysplastic colon tissue 70%, Immune cells 30%
7	Necrosis	Avital tumor with necrosis	Tiles are approx. half background, some stroma and necrosis, but mostly moderately differentiated tumor	Mostly moderatly differentiated tumor epithelium	Some strands of aligned tumor stroma, stroma-low tiles	Some immune cells and necrosis, immune-high	Much background	Tumor epithelial cells 40%, Background 30%, Stroma 10%, Necrosis 20%
8	Tumor epithelium	Mixed tumor epithelial - infiltrated stroma	Some variation, mostly tumor and some stroma in different levels of differentiation and organization.	Mostly moderatly differentiated tumor epithelium	Some stroma on mostly stroma-low tiles, some aligned strands but more disorganized in between tumor epithelium	Part immune cells, immune-high	Some background and spots of erythrocytes	Tumor epithelial cells 40%, Stroma 20%, Immune cells 20%, Background 10%, Erythrocytes 10%
9	Tumor epithelium	Moderately differentiated tumor - aligned stroma	Some background, but mostly moderately differentiated tumor and some stroma in between, a few spots of mucin	Mostly well-moderately differentiated tumor epithelium	Some aligned strands of stroma, tiles are stroma-low	A few immune cells, immune-low	Some background, some mucin	Tumor epithelial cells 70%, Stroma 20%, Background 10%
10	Muscle tissue	Muscle tissue (longitudinal fibers)	Muscle tissue fiber strands as well as similar looking aligned strands of stroma. Some vessels with erythrocytes, and immune cells in between	No tumor epithelium	Some tiles contain strands of aligned stroma, then the tile is stroma-high	Some immune cells, immune-low in stroma tiles	Mostly muscle tissue, longitudinal fibers and some vessels	Muscle 60%, Stroma 30%, Immune cells 10%
11	Tumor stroma	Vascularized and infiltrated stroma	Mostly much tumor stroma with neovascularization, and with many immune cells in between, potentially parts of lymphoid tissue. Few isolated tumor epithelial cells	Few isolated tumor epithelial cells	Mostly much and disorganized tumor stroma with neovascularization, stroma-high	High amount of immune cells, potentially part of lymphoid tissue. Immune-high	A couple tiles with a bit background or fatty tissue	Stroma 70%, Immune cells 30%
12	Mucinous	Mucinous tumor	Some background in otherwise mucinous tumor containing tiles. Notably high contrast in colors, little to no stroma	Predominantly mucinous tumor epithelium	Some stroma, disorganized spots. Stroma-low	Some immune cells, immune-low	Mostly mucus, some background and one tile with fatty tissue	Tumor epithelial cells 40%, Mucin 30%, Background 20%, Stroma 10%
13	Immune cells	Infiltrated stroma	The mostly aligned tumor stroma is highly infiltrated with immune cells, with some well-moderately differentiated tumor epithelial tissue and some background	Well differentiated tumor epithelium, high-grade dysplasia	Mostly aligned, straight strands of tumor stroma on the stroma-low tiles	High amount of immune cells in stroma, potentially part of lymphoid tissue. Also some dirty necrosis. Immune-high	N/A	Immune cells 30%, Stroma 30%, Tumor epithelial cells 30%, Necrosis 10%
14	Mucinous	Mucinous tumor	Tumor epithelial cells with some stroma, on tiles consisting approx. half of mucin, some background	Predominantly mucinous tumor epithelium	A part disorganized tumor stroma, some aligned strands. Officially stroma-high tiles, excluding the mucus	A few immune cells, immune-low	Mostly mucus, some background	Mucin 40%, Tumor epithelial cells 30%, Stroma 20%, Background 10%
15	Fatty tissue	Fatty tissue	Much fatty tissue, some vessels (also round shape), strands of mostly aligned stroma, some immune cells	No tumor epithelium	Strands of aligned stroma, potentially not only tumor-induced but some healthy as well	A part immune cells, some lymphoid tissue, in stroma then immune-high overall	Predominantly fatty tissue on the tiles, and some other round shapes like vessels, some with erythrocytes, and a spot of mucin	Fatty tissue 60%, Stroma 30%, Immune cells 10%
16	Tumor epithelium	Well-differentiated tumor	Mostly well-moderately differentiated tumor, some stroma in between, and dirty necrosis	Well differentiated tumor epithelium, high-grade dysplasia	Some aligned strands of stroma, tiles are stroma-low	Some immune cells, some dirty necrosis, immune-low	Some background	Tumor epithelial cells 60%, Stroma 20%, Necrosis 10%, Background 10%

HPC	Super-cluster	Label	General description	Tumor epithelium	Tumor stroma	Immune cells	Other	HPC tissue type composition
**17**	Tumor stroma	Infiltrated aligned stroma-high	Some immune cells and tumor epithelial cells in the much most prevalent aligned strands of stroma (stroma-high). Sometimes a neural bundle and a few spots more similar to muscle fibers	Some parts tumor epithelium, well-differentiated	Predominantly tumor stroma, mostly aligned strands on stroma-high tiles	Part immune cells infiltrated in tumor stroma, immune-high overall	Some spots background, a couple tiles with some muscle tissue. A sporadic neural bundle	Stroma 60%, Immune cells 20%, Tumor epithelial cells 10%, Muscle tissue 10%
**18**	Immune cells	Infiltrated stroma	Cluster abundant in cells. Much stroma, mostly disorganized, with many immune cells and some isolated tumor cells	Part isolated tumor epithelial cells	Predominantly tumor stroma, mostly disorganized stroma-high tiles	Many immune cells infiltrated in tumor stroma, immune-high	N/A	Stroma 60%, Immune cells 30%, Tumor epithelial cells 10%
**19**	Tumor stroma	Edematous disorganized stroma	Much edematous, mostly disorganized stroma, almost no immune cells but with tumor epithelium, some necrosis	Some parts tumor epithelium, well-differentiated	Much edematous tumor stroma, mostly disorganized stroma-high tiles	Few immune cells, some (dirty) necrosis, immune-low	A few spots mucin	Stroma 70%, Tumor epithelial cells 30%
**20**	Tumor epithelium	Disorganized and vascularized stroma-low	Some background but mostly moderately differentiated tumor epithelial cells in disorganized stroma, some immune cells	Well-moderately differentiated tumor	Mostly disorganized stroma, stroma-low tiles, some vascularization	A part immune cells, some (dirty) necrosis, immune-low mostly	Some background and a few spots mucin, a slight signet cell component	Tumor epithelial cells 50%, Stroma 30%, Immune cells 10%, Background 10%
**21**	Tumor stroma	Vascularized stroma	Aligned strands of much loose stroma with neovascularization (or small white shapes similar to vessels), sometimes some muscle fibers, with immune cells and isolated tumor cells in between	A couple isolated tumor cells	Predominantly aligned tumor stroma strands, stroma-high, vascularized (or other round shapes)	Part immune cells infiltrated in tumor stroma, some parts immune-high	Some background, a few spots of fatty tissue. A couple tiles muscle tissue in stead of stroma	Stroma 70%, Immune cells 10%, Tumor epithelial cells 10%, Muscle tissue 10%
**22**	Immune cells	Infiltrated stroma	Many immune cells in tumor-induced and healthy stroma, with some tumor or healthy colon tissue and some background	Some well differentiated tumor epithelium, high-grade dysplasia	Healthy and tumor stroma, which is stroma-high	High number of immune cells, some necrosis, immune-high	Some healthy and dysplastic tissue	Immune cells 30%, Stroma 30%, Tumor epithelial cells 20%, Background 10%, Healthy and dysplastic colon tissue 10%
**23**	Healthy and dysplastic colon tissue	Dysplastic colon tissue	Mostly dysplastic colon tissue (adenoma), with many immune cells in between and much background and mucin	A couple isolated tumor cells	Dysplastic stroma	Many immune cells in dysplastic stroma	Predominantly dysplastic colon tissue, some background and mucin	Healthy and dysplastic colon tissue 40%, Immune cells 20%, Mucin 20%, Background 20%
**24**	Muscle tissue	Vessel-like stroma/ muscle	Approx. half of the tiles contain background, some mucin, the other half is stroma and/or muscle tissue of vessels; large, white round shapes. There is some dirty necrosis	A couple isolated tumor cells	Stroma in the shape of vessels, these are mostly aligned tumor stroma and stroma-high	A few immune cells, some dirty necrosis, immune-low	Muscle tissue, mostly the walls from vessels and background, a spot of erythrocytes within these vessles, some mucin	Muscle tissue 30%, Stroma 30%, Background 30%, Mucin 10%
**25**	Necrosis	Moderately differentiated tumor - necrotic	Moderately differentiated tumor epithelial with (dirty) necrosis, and some stroma in between. Some background and mucin as well	Mostly well differentiated tumor epithelial cells	Some strands of aligned tumor stroma, stroma-low tiles	Part immune cells in (dirty) necrosis, otherwise immune-low	In general busy and disorganized tiles, round shapes, some background, some mucin	Tumor epithelial cells 50%, Necrosis 20%, Stroma 10%, Mucin 10%, Background 10%
**26**	Tumor epithelium	Infiltrated poor-undifferentiated tumor	Cluster rich in cells. Tiles with solid growing poor-undifferentiated tumor, others immune cells and some stroma in between. Potentially microsatellite instability	Tiles with solid growing poor-undifferentiated tumor epithelium	A couple strands of aligned tumor stroma, stroma-low tiles	Many immune cells, some tiles only containing immune cells, immune-high	Cluster rich in cells, one tile a slight signet cell component, a spot background	Tumor epithelial cells 80%, Immune cells 10%, Stroma 10%
**27**	Tumor epithelium	Moderately differentiated tumor - aligned stroma	Some background or mucin. Well-moderately differentiated tumor epithelial cells in some aligned strands of stroma and some immune cells	Well differentiated tumor epithelium, high-grade dysplasia	Some strands of aligned tumor stroma, stroma-low tiles	Some immune cells, also in (dirty) necrosis, overall immune-high	Some background	Tumor epithelial cells 50%, Stroma 30%, Immune cells 10%, Background 10%
**28**	Muscle tissue	Muscle (longitudinal fibers)	Mostly muscle tissue, longitudinal sections, sometimes aligned stroma strands and some background. A few tumor epithelial cells and neural bundles	A couple isolated tumor cells	A couple tiles contain strands of aligned stroma, these are stroma-high	A few immune cells, immune-low in stroma tiles	Predominantly muscle tissue in longitudinal fibers, some background or a spot of fatty tissue. A sporadic neural bundle	Muscle tissue 70%, Stroma 20%, Background 10%
**29**	Muscle tissue	Muscle (longitudinal fibers)	Mostly longitudinal sectioned muscle tissue and/or stroma strands, a bit edematous, some vessels, a neural bundle, not many immune cells	A couple isolated tumor cells	A couple tiles contain strands of aligned stroma, then the tile is stroma-high	A few immune cells, immune-low in stroma tiles	Predominantly muscle tissue in longitudinal fibers, some background or a spot of fatty tissue. A sporadic neural bundle. Small vessels often in between	Muscle tissue 80%, Stroma 20%
**30**	Immune cells	Mixed, immune-high	Aligned stroma strands and well-moderately differentiated tumor epithelial cells, immune cells in between, some dirty necrosis	Well differentiated tumor epithelium, high-grade dysplasia	Aligned stroma strands on mostly stroma-low tiles	Many immune cells infiltrated in tumor stroma, some spots of (dirty) necrosis	N/A	Tumor epithelial cells 30%, Stroma 30%, Immune cells 30%, Necrosis 10%
**31**	Immune cells	Mixed, infiltrated stroma	Often aligned stroma with many immune cells and moderately differentiated tumor epithelium. Often some background, some necrosis	Well differentiated tumor epithelium, high-grade dysplasia	Aligned, loose stroma strands on mostly stroma-low tiles	Part immune cells often infiltrated in tumor stroma, immune-high	Some background, a couple spots of mucin or fatty tissue	Stroma 40%, Immune cells 20%, Tumor epithelial cells 20%, Background 20%

HPC	Super-cluster	Label	General description	Tumor epithelium	Tumor stroma	Immune cells	Other	HPC tissue type composition
32	Muscle tissue	Muscle (axial section)	Axial sections of muscle tissue, some disorganized strands of stroma and some background. Also, some tiles that contain erythrocytes, similar looking to the muscle fiber sections	No tumor epithelium	No tumor stroma	A few immune cells	Tiles contain mostly muscle tissue, with the fibers in axial sections. Some are part of a vessel wall, with sometimes also erythrocytes in vessels. Some background	Muscle tissue 70%, Erythrocytes 10%, Stroma 10%, Background 10%
33	Muscle tissue	Muscle (longitudinal fibers)	Muscle fibers in longitudinal sections or aligned stroma strands, with much background and fatty tissue, some vessels and only few immune cells	A couple isolated tumor cells	Some tiles contain strands of aligned stroma, then the tile is stroma-high	A few immune cells, immune-low in stroma tiles	Some background, some fatty tissue, in between loose bundles of longitudinal muscle fibers, some muscle tissue as part of vessels	Muscle tissue 40%, Stroma 30%, Background 20%, Fatty tissue 10%
34	Muscle tissue	Muscle (axial section)	Mostly muscle tissue in axial plane, some tiles disorganized tumor stroma, with some vessels; overall few nuclei or immune cells	A couple isolated tumor cells	A couple tiles contain strands of disorganized tumor stroma, these are stroma-high	A few immune cells, immune-low in stroma tiles	Predominantly axial sections of muscle tissue, a few spots of background or mucus	Muscle tissue 70%, Stroma 30%
35	Immune cells	Immune cells	Mostly immune cells in stroma or lymphoid tissue, with some tumor epithelial cells or necrosis	A couple isolated tumor cells	Tumor- and healthy stroma, stroma-high tiles	Highly infiltrated (tumor) stroma or necrosis, sometimes part of lymphoid tissue, immune-high	Some background, a few spots of mucin or fatty tissue, a few spots of dysplastic colon tissue	Immune cells 60%, Stroma 20%, Tumor epithelial cells 10%, Background 10%
36	Necrosis	Avital tumor with necrosis	Loose and pieces of tissue, mostly avital tumor epithelium or necrosis, in tiles with much background, some strands of stroma	Loose and pieces of tissue, mostly avital tumor epithelium, well-moderately differentiated	Some spots of stroma, mostly disorganized, stroma-low tiles	Immune cells in much present necrosis	Much background, a few spots of mucin	Necrosis 30%, Tumor epithelial cells 30%, Background 30%, Stroma 10%
37	Healthy and dysplastic colon tissue	High-grade dysplasia - adenoma	Adenoma or well differentiated tumor, with much background, some mucin, and few spots of dirty necrosis	Adenoma, high-grade dysplasia or well differentiated tumor epithelium	A few strands of aligned (tumor) stroma, stroma-low	Some immune cells in dysplastic and tumor stroma, a spot of dirty necrosis	Some background and/or mucin	Healthy or dysplastic colon tissue 60%, Stroma 10%, Immune cells 10%, Background 10%, Mucin 10%
38	Mucinous	Mucinous tumor stroma	Mucin, mostly from tumors with disorganized tumor stroma, some background	A couple isolated tumor cells	Some disorganized strands of tumor stroma in between the mucin, tiles are stroma-high	A few immune cells, immune-low	Predominantly mucin	Mucin 80%, Stroma 20%
39	Healthy and dysplastic colon tissue	Healthy colon tissue	Mostly clear, healthy colon tissue with mucin in goblet cells and the villi, a small component dysplasia, some background and immune cells in between	No tumor epithelium	No tumor stroma	Some immune cells in healthy stroma, normal	Predominantly healthy colon tissue, some background and/or mucin	Healthy or dysplastic colon tissue 80%, Background 10%, Mucin 10%
40	Tumor stroma	Disorganized stroma, growing infiltratively	Polymorphic and poorly differentiated tumors in much prevalent edematous and disorganized stroma, growing infiltratively, a few immune cells	Part polymorphic and poorly differentiated tumor epithelium, growing infiltratively	Much prevalent edematous and disorganized tumor stroma, often vascularized, stroma-high	Part immune cells, immune-low	N/A	Stroma 60%, Tumor epithelial cells 30%, Immune cells 10%
41	Tumor stroma	Disorganized and loose stroma	Heterogeneous cluster. Some background and/or (loose) stromal tissue, not all tumor-associated, with some vessels and immune cells, a few spots of mucin	A couple isolated tumor cells	Predominantly loose aligned stroma fibers, some tumor-induced, then stroma-high. Vascularized	Part immune cells, immune-low	Some tiles contain muscle tissue from vessels, some background, a few spots of mucin	Stroma 70%, Muscle tissue 10%, Background 10%, Immune cells 10%
42	Tumor epithelium	Moderately differentiated tumor	Mostly moderately differentiated tumor epithelial with a few aligned strands of stroma in between. Some background and a few artefacts	Mostly well-moderately differentiated tumor epithelium	Some aligned strands of stroma, tiles are stroma-low	Few immune cells, some spots (dirty) necrosis, immune-low	Some background	Tumor epithelial cells 70%, Stroma 20%, Background 10%
43	Necrosis	Necrotic	Necrosis with anucleic cells, or fields with mostly immune cells. Much background and some tumor epithelial cells	Some moderately tumor epithelium, some isolated tumor epithelial cells	A few strands of aligned (tumor) stroma	Immune cells in much present necrosis	Some background	Necrosis 50%, Immune cells 20%, Background 20%, Tumor epithelial cells 10%
44	Tumor stroma	Disorganized, immune-low stroma (desert-type)	Mostly aligned stroma, desert type with few nuclei and immune cells, potentially some not tumor-induced with a few isolated tumor epithelial cells	A couple isolated tumor cells	Much prevalent edematous and disorganized tumor stroma, often vascularized, stroma-high	A few immune cells, immune-low	A couple tiles contain longitudinal muscle fibers	Stroma 90%, Immune cells 10%
45	Tumor epithelium	Poor-undifferen-tiated tumor	Poor-undifferentiated tumor epithelium, with some disorganized stroma, some background	Much poor-undifferentiated tumor epithelium	Some disorganized tumor stroma, stroma-low	A few immune cells, immune-low	Some background	Tumor epithelial cells 70%, Stroma 20%, Background 10%
46	Tumor epithelium	Infiltrated undifferen-tiated tumor	Cluster rich in cells. Solid growing, undifferentiated tumor, with some immune cells. Potentially micro-satellite instability. Component signet cell carcinoma and squamous cells	Much poor-undifferentiated tumor epithelium	A few strands of aligned (tumor) stroma, stroma-low	Many immune cells, also a couple tiles with only immune cells, immune-high	A tile with slight component signet cell, one with necrosis	Tumor epithelial cells 70%, Immune cells 20%, Stroma 10%

Supplementary Table 1: The table shows all HPCs with tissue compositions, including labels, super-clusters, and detailed description of aspect and percentage of tissue types. HPC, histomorphological phenotype cluster. N/A, not applicable.

Supplementary Table 2: Summary of the associations between HPCs and overall survival

HPC	Association	Treatment group	Potential associations with OS	References
**0**	Worse survival	TCGA and AVANT-standard	Disorganized stroma indicates active remodeling and an intense, yet failing, host response to aggressive invasive tumor cells. The tiles are also stroma-high, consistent with results from the immune landscape analysis, also leading to a worse survival.	Mesker et al (2007), Friedl et al (2011), Zunder et al (2018), Zunder et al (2020), Ueno et al (2021), Strous et al (2022), Polack et al (2024)
**2**	Better survival	TCGA and AVANT-standard	Aligned stroma indicates a successful defense of the host against less invasive tumor cells. Moreover, the tiles are mostly stroma-low with well-differentiated tumor epithelium leading to a better survival.	Mesker et al (2007), Friedl et al (2011), Weiser et al (2011), Ueno et al (2012), Zunder et al (2018), Zunder et al (2020), Ueno et al (2021), Strous et al (2022), Polack et al (2024)
**3**	Better survival	TCGA and AVANT-standard	Well-to-moderately differentiated (low-grade) tumor epithelium, along with some high-grade dysplasia and low levels of stromal contents, are well-established indicators of a better survival.	Mesker et al (2007), Weiser et al (2011), Ueno et al (2012), Brierley et al (2016), Weiser et al (2018), Zunder et al (2018), Strous et al (2022), Polack et al (2024)
**4**	Better survival	AVANT-experimental	This cluster consists mostly of well-to-moderately differentiated (low-grade) tumor epithelium, with stroma-low tiles and an influx of immune cells, which is associated with a favorable survival.	Mesker et al (2007), Weiser et al (2011), Ueno et al (2012), Brierley et al (2016), Pages et al (2018), Weiser et al (2018), Zunder et al (2018), Bagaev et al (2021), Strous et al (2022), Polack et al (2024)
**5**	Better survival	AVANT-experimental	Much necrosis, promoting the expression of VEGFa as target of bevacizumab, is present in this cluster, leading to a better response to bevacizumab. Enrichment in pathways of cell cycle and DNA repair may also indicate more targets to the cytotoxic chemotherapy and promote survival.	Ferrara et al (2002), Hurwitz et al (2004), Kap et al (2015), Roos et al (2016), Vodicka et al (2019)
**6**	Worse survival	TCGA and AVANT-standard	This cluster contains a significant amount of inflamed and partially dysplastic colon tissue, indicating a large amount of remodeling. The presence of potential chronic inflammation predisposes the development of cancer as well as gives rise to a possible cancer field effect, hence leading to a worse survival.	Ullman et al (2011), Lockhead et al (2015), Brierley et al (2016), Weiser et al (2018)
**7**	Better survival	TCGA and AVANT-standard	Avital tumor and necrosis in this cluster are signs of poor neovascularization and thus potentially indicating an less aggressive tumor. Additionally, downregulation in epithelial-to-mesenchymal transition pathways could lead to favorable outcomes.	Ferrara et al (2002), Brierley et al (2016), Weiser et al (2018), Sandberg et al (2019)
**9**	Better survival	AVANT-experimental	Besides relatively favorable histopathology aspects similar to some previous HPCs (e.g. HPC 4, low-grade tumor epithelium), downregulation of pathways related to epithelial-to-mesenchymal transition and angiogenesis, as well as lower tumor necrosis factor-alpha could lead to an improved survival.	Ferrara et al (2002), Zins et al (2007), Weiser et al (2011), Ueno et al (2012), Weiser et al (2018), Sandberg et al (2019)
**11**	Worse survival	TCGA and AVANT-standard	Neovascularization in stroma indicates active host response with wound healing features, known to have tumor promoting properties. Tiles are stroma-high and linked to an epithelial-to-mesenchymal transition in the gene set enrichment analysis, associated to worse outcomes.	Ferrara et al (2002), Mesker et al (2007), Zunder et al (2018), Sandberg et al (2019), Strouss et al (2022), Polack et al (2024)
**12**	Worse survival	AVANT-experimental	Mucinous tumors are well-known to lead to worse prognosis and can be linked to a higher mutational burden. *KRAS* signaling-up pathways and depletion in cell cycle regulatory pathways such as DNA repair, overall lead to worse survival.	Kap et al (2015), Brierley et al (2016), Chang et al (2016), Roos et al (2016), Shia et al (2017), Weiser et al (2018), Nagtegaal et al (2019), Vodicka et al (2019), Zhu et al (2021)
**13**	Better survival	TCGA and AVANT-standard	The immune cell influx has a positive correlation with survival, indicating a good host response; an increased leukocyte fraction was seen in the immune landscape analysis as well.	Pages et al (2018), Bagaev et al (2021)
**14**	Worse survival	AVANT-experimental	Similar to HPC 12, mucinous tumors are well-known to lead to a worse prognosis and can be linked to a higher mutational burden. *KRAS* signaling-up pathway and depletion in cell cycle regulatory pathways such as DNA repair, overall lead to worse survival.	Kap et al (2015), Brierley et al (2016), Chang et al (2016), Roos et al (2016), Shia et al (2017), Weiser et al (2018), Nagtegaal et al (2019), Vodicka et al (2019), Zhu et al (2021)

HPC	Association	Treatment group	Potential association with OS	References
**17**	Worse survival (TCGA and AVANT-standard) AND Better survival (AVANT-experimental)	Both treatment groups	Mixed associations to survival are seen here. This could be due to high amount of stroma and enrichment in epithelial-to-mesenchymal transition gene pathways, which are poor prognostic factors and lead to a worse survival among the standard-treated patients. However, both the aligned stroma with immune infiltration and the enrichment in the hypoxia pathway (involving the expression of VEGFa gene as the target of bevacizumab), lead to a better survival in experimentally-treated patients.	Ferrara et al (2002), Hurwitz et al (2004), Mesker et al (2007), Friedl et al (2011), Pages et al (2018), Zunder et al (2018), Sandberg et al (2019), Zunder et al (2020), Bagaev et al (2021), Ueno et al (2021), Strouss et al (2022), Polack et al (2024)
**18**	Better survival	AVANT-experimental	The immune cell component may indicate an effective response from the host and thus lead to a favorable survival. Enrichment in the angiogenesis pathway (involving VEGFa as the target of bevacizumab) may indicate an improved survival in patients receiving bevacizumab.	Hurwitz et al (2004), Kap et al (2015), Pages et al (2018), Bagaev et al (2021)
**19**	Worse survival	TCGA and AVANT-standard	Stroma-high tiles categorized by disorganized stroma, including an absence of immune cells, indicate an active remodeling with a failing host response, which collectively may lead to a poor survival outcome.	Friedl et al (2011), Kap et al (2015), Pages et al (2018), Zunder et al (2018), Zunder et al (2020), Bagaev et al (2021), Ueno et al (2021), Strouss et al (2022), Polack et al (2024)
**20**	Worse survival	AVANT-experimental	Disorganized stroma can be a sign of insufficient host response and matrix remodeling. Enrichment in pathways of *KRAS* signaling-up and hypoxia, along with depletion in cell cycle regulation pathways, may contribute to an unfavorable survival through several plausible mechanisms.	Friedl et al (2011), Kap et al (2015), Chang et al (2016), Roos et al (2016), Vodicka et al (2019), Zunder et al (2020), Ueno et al (2021), Zhu et al (2021)
**21**	Worse survival	TCGA and AVANT-standard	Neovascularization in stroma-high tiles, as indicator of the failure of host response, and enrichment in oncogenic pathways may cause an unfavorable survival in the standard-treated patients.	Ferrera et al (2002), Kap et al (2015), Pages et al (2018), Bagaev et al (2021)
**23**	Worse survival	TCGA and AVANT-standard	Tiles are characterized by precancerous dysplastic colon tissue and chronic inflammation, both of which predispose to cancer development and suggest a cancer field effect, potentially leading a worse survival.	Ullman et al (2011), Lockhead et al (2015), Brierley et al (2016), Weiser et al (2018)
**24**	Better survival (AVANT-standard) AND Worse survival (AVANT-experimental)	Both treatment groups	The underlying mechanisms of the mixed results between the standard and experimental treatment groups remains unclear. However, we hypothesize that this may be due to the complex interactions between mixed histopathological patterns and gene expression patterns. On one hand, the worse survival may be attributed to the stroma-high histopathological features of stromal strands in the muscle fibers.<br>This is further confirmed by the upregulation of epithelial-to-mesenchymal transition pathway that has been linked to a poor survival. Enrichment in *KRAS* signaling-up and depletion cell cycle regulatory pathways may be associated with a worse response to chemotherapy. On the other hand, enrichment in interferon-gamma response and inflammatory response pathways may indicate a strong host immune response which may lead to a better survival.	Ferrera et al (2002), Hurwitz et al (2004), Zins et al (2007), Friedl et al (2011), Kap et al (2015), Chang et al (2016), Roos et al (2016), Pages et al (2018), Sandberg et al (2019), Vodicka et al (2019), Jorgovanovic et al (2020), Zunder et al (2020), Bagaev et al (2021), Zhu et al (2021), Polack et al (2024)
**25**	Worse survival	Both treatment groups	Necrotic and mucinous components may be associated with a worse survival in general. We also observed depletion in expression of genes in several immune related pathways (e.g. interferon-gamma response). In addition, no enrichment was observed in the pathways involving VEGFa, potentially indicating a low number of targets for bevacizumab.	Ferrara et al (2002), Brierley et al (2016), Pages et al (2018), Weiser et al (2018), Nagtegaal et al (2019), Jorgovanovic et al (2020), Bagaev et al (2021)
**26**	Better survival	AVANT-experimental	Tiles are high in immune cells despite the presence of poorly-to-undifferentiated (high-grade) tumor epithelium, which has been linked to tumors with microsatellite instability. This is known to lead to a better survival. Enrichment in cell cycle regulatory pathways may indicate more targets for cytotoxic chemotherapy, potentially enhancing the survival.	Boland et al (2010), Brierley et al (2016), Shia et al (2017), Pages et al (2018), Weiser et al (2018), Nagtegaal et al (2019), Bagaev et al (2021)

HPC	Association	Treatment group	Potential association with OS	References
**27**	Better survival	AVANT-experimental	Aligned stroma and influx of immune cells are both predictors for a good response to cytotoxic chemotherapy. Enrichment in the hypoxia pathway, which involves the VEGFa gene, potentially indicate more targets and a better response to bevacizumab. Together, these factors may result in an improved outcome in the experimentally treated patients.	Hurwitz et al (2004), Pages et al (2018), Kap et al (2015), Bagaev et al (2021), Ueno et al (2021)
**28**	Better survival	Both treatment groups	Higher fractions of this muscle tissue-abundance cluster may indicate more healthy tissue, which may lead a better prognosis. No tumor buds was observed (in contrast to HPC 33), which may indicate a less aggressive tumor.	Brierley et al (2016), Lugli et al (2017), Weiser et al (2018), Nagtegaal et al (2019)
**31**	Better survival	Both treatment groups	Aligned stroma and an immune component are both favorable factors regarding response to cytotoxic therapy. This is consistent with the high leukocyte fraction observed in the immune landscape analysis. Moreover, enrichment in pathways of hypoxia and angiogenesis, which involves VEGFa, may indicate more targets for bevacizumab. Enrichments in cell cycle regulation (e.g. G3/M checkpoint, mTORC1) may suggest more targets for the chemotherapy.	Ferrara et al (2002), Hurwitz et al (2004), Roos et al (2016), Pages et al (2018), Vodicka et al (2019), Zunder et al (2020), Bagaev et al (2021), Ueno et al (2021)
**33**	Worse survival	AVANT-experimental	The worse survival may be related to tumor buds invading the serosa and fatty tissue, a known indicator for aggressive tumors. Fatty tissue is also known as a cancer inducer, providing energy for cancer cells. The relative absence of immune cells observed in the tiles suggests a weak host response, which is further reflected in the depletion in inflammatory and angiogenesis pathways. Together, these factors may contribute to poor survivals.	Ferrara et al (2015), Lugli et al (2017), Pages et al (2018), Bagaev et al (2021), Brown et al (2023)
**34**	Better survival	AVANT-experimental	Muscle tissue abundance may suggest fractionally more healthy tissue, which in turn suggests a smaller tumor size and thus a better prognosis. Moreover, we observed neovascularization in the tiles, which in the experimentally-treated group could enhance survival. Enrichment in pathways related to cell cycle regulations may indicate more targets to the cytotoxic chemotherapy.	Ferrera et al (2002), Kap et al (2015), Brierley et al (2016), Roos et al (2016), Weiser et al (2018), Nagtegaal et al (2019), Vodicka et al (2019)
**38**	Better survival (AVANT-standard) AND Worse survival (AVANT-experimental)	Both treatment groups	Mechanisms underlying these different associations remain unclear. On one hand, mucinous tumors are known to lead to a worse prognosis. *KRAS* signaling-up pathways and depletion in cell cycle regulatory pathways may suggest a poor response to chemotherapy. On the other hand, enrichment in several inflammatory response pathways may suggest a strong host immune response that results in a better survival.	Kap et al (2015), Brierley et al (2016), Chang et al (2016), Roos et al (2016), Pages et al (2018), Weiser et al (2018), Nagtegaal et al (2019), Vodicka et al (2019), Jorgovanovic et al (2020), Bagaev et al (2021), Zhu et al (2021)
**39**	Better survival	Both treatment groups	Tiles contain healthy colon tissue. Higher fraction of healthy colon tissue tiles may indicate relatively smaller tumor size and potentially less cancer field effect in a larger tissue area (i.e. little disturbances in the healthy tissue) and leads to a better survival.	Lockhead et al (2015), Brierley et al (2016), Weiser et al (2018), Nagtegaal et al (2019)
**40**	Worse survival	Both treatment groups	Disorganized stroma, indicating an insufficient host response, and poor-to-undifferentiated (high-grade) tumor epithelium could both lead to an unfavorable survival. A cancer field effect negatively influences surrounding tissue, adding to the poor association. Depletion of cell cycle pathways may suggest few targets for cytotoxic chemotherapy and thus worse outcomes.	Friedl et al (2011), Weiser et al (2011), Ueno et al (2012), Kap et al (2015), Lockhead et al (2015), Roos et al (2016), Nagtegaal et al (2019), Vodicka et al (2019), Zunder et al (2020), Ueno et al (2021)
**41**	Worse survival	AVANT-experimental	The combination of disorganized and loose stroma as well as absence of immune components indicates active remodeling and failure of the immune response. Depletion in cell cycle pathways may suggest fewer targets for cytotoxic chemotherapy and result in a worse survival.	Friedl et al (2011), Kap et al (2015), Roos et al (2016), Vodicka et al (2019), Zunder et al (2020), Bagaev et al (2021), Ueno et al (2021)
**45**	Worse survival	TCGA and AVANT-standard	Poor-to-undifferentiated (high-grade) tumor epithelium is a well-known indicator of poor survival.	Weiser et al (2011), Ueno et al (2012), Brierley et al (2016), Weiser et al (2018), Nagtegaal et al (2019)
**46**	Better survival	TCGA and AVANT-standard	Abundance in immune cell components is positively correlated with a better survival. Poorly-to-undifferentiated (high-grade) tumor epithelium together with enrichment in immune-related gene pathways may potentially indicate microsatellite instability which is known to indicate a better survival.	Boland et al (2010), Brierley et al (2016), Shia et al (2017), Pages et al (2018), Weiser et al (2018), Nagtegaal et al (2019), Bagaev et al (2021)

Supplementary Table 2: **The table summarizes HPCs that were significantly associated with OS per treatment group, including references.** Color legend: Orange, HPCs associated with worse survival in one group. Red, HPCs associated with worse survival in both groups. Blue, HPCs associated with improved survival in one group. Green, HPCs associated with improved survival in both groups. Grey, HPCs with mixed associations with OS in the two treatment groups. AVANT, Bevacizumab-Avastin® adjuVANT trial. HPC, histomorphological phenotype cluster. OS, overall survival. TCGA, The Cancer Genome Atlas. VEGF, vascular endothelial growth factor.

a

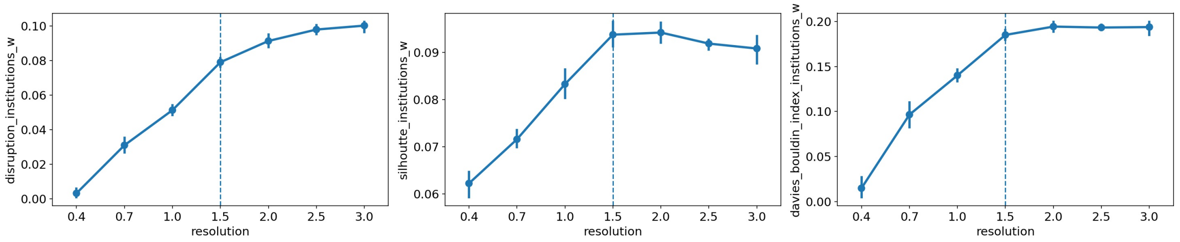

Leiden resolution optimization using unsupervised methods in TCGA

b

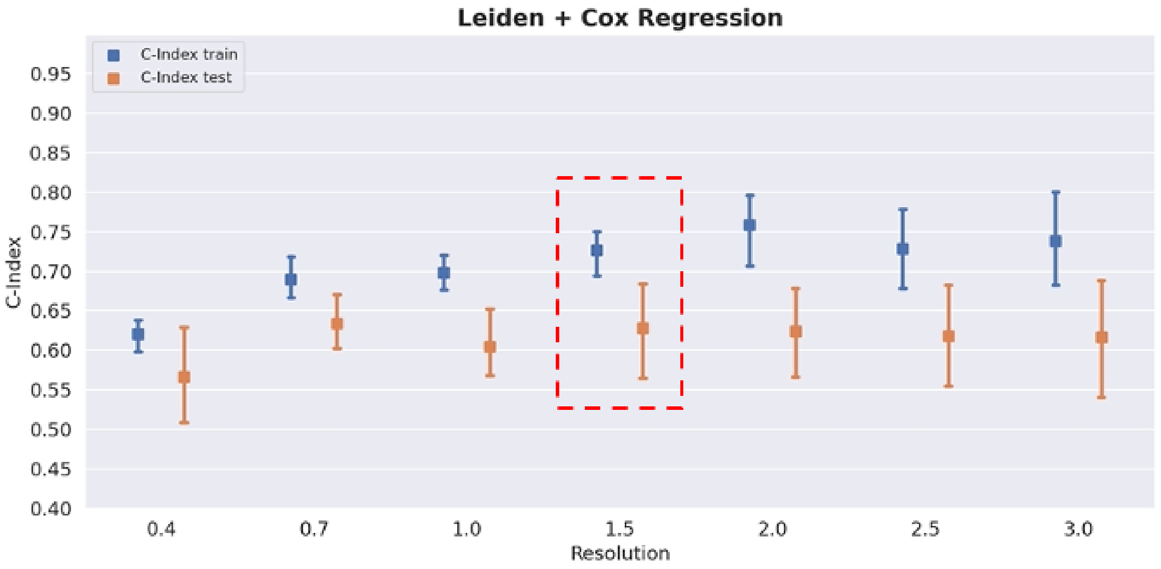

Leiden resolution optimization on OS prediction from regularized Cox regression in TCGA

Supplementary Figure 1: **Optimization of Leiden resolution** (a) Disruption score, Silhouette score, and Daves-Boundin index weighted by mean percentage of the institution presence in each HPC reached a consensus on optimal Leiden resolution at 1.5 in TCGA (n=405 patients). (b) We trained regularized Cox regressions including all 47 HPCs for each Leiden resolution using 5-fold CV in TCGA (n=405 patients). The Leiden resolution 0.7 and 1.5 showed the highest validation c-index. We chose Leiden 1.5 as the optimal resolution because it led to more explorable HPCs. CV, cross-validation. HPC, histomorphological phenotype cluster. OS, overall survival. TCGA, The Cancer Genome Atlas. n, number of patients included in the analyses after those with missing clinical data. Source data are provided as a Source Data file.

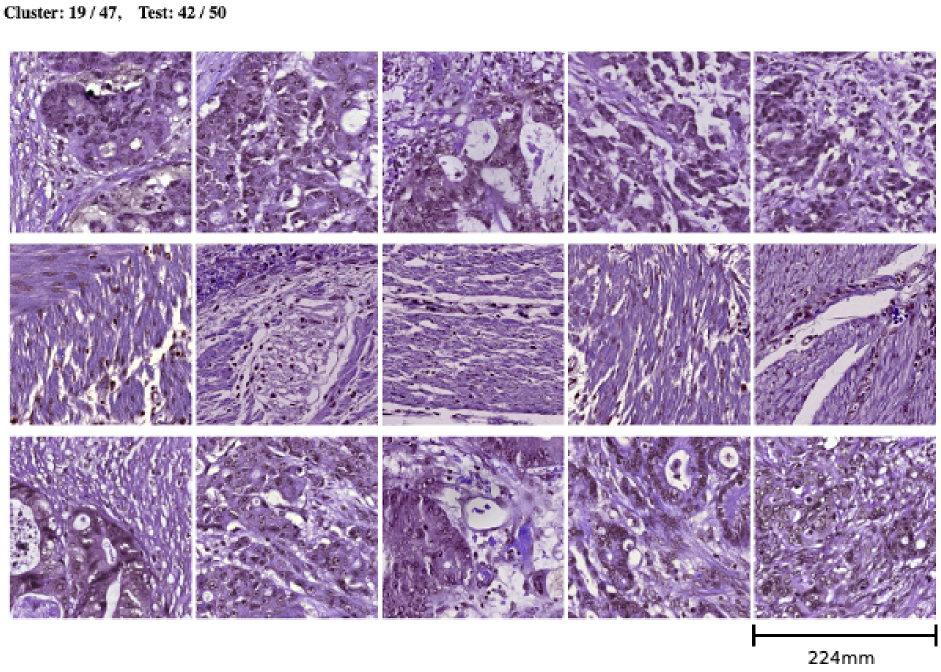

Supplementary Figure 2: **Objective test for all HPCs.** Screenshot of the online objective test. Three rows of tiles were presented at each test, with 2 rows were from the same HPC and the other row from a randomly selected different HPC. The image tiles (224-by-224 pixels), at a magnification level of 10x (pixel size approximate 1.0 um), correspond to 224mm in size (see scale bar)

a HPC-based classifier in five-fold CV in TCGA

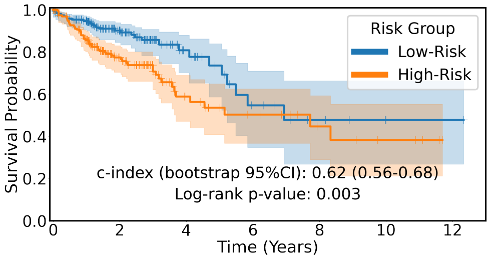

b HPC-based classifier in the external AVANT control group

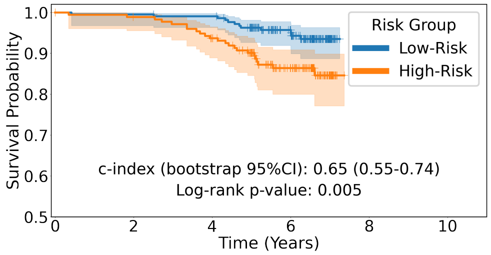

c Clinical baseline classifier in the external AVANT control group

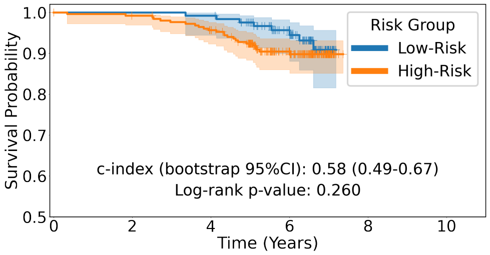

d HPC-based classifier in five-fold CV in AVANT-experimental group

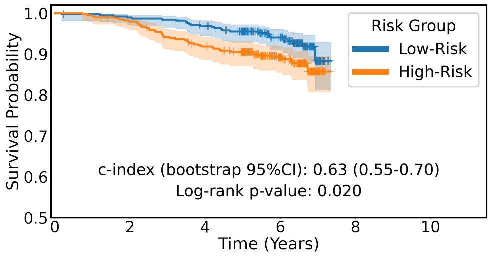

Supplementary Figure 3: Regularized Cox regression for OS prediction. (a) Kaplan-Meier plot showing the HPC-based classifier stratifying OS risk in the five-fold CV in TCGA (two-sided log-rank p=0.003, n=405 patients). (b) The HPC-based classifier stratifying OS risk in the external AVANT control test set (two-sided log-rank p=0.005, n=391 patients). (c) The clinical baseline model (predictors including age, sex, tumor-stroma ratio, AJCC TNM staging) stratifying OS risk in the independent AVANT control test set (two-sided log-rank p=0.260, n=378 patients). (d) The HPC-based classifier stratified OS risk AVANT-experimental group (two-sided log-rank p=0.020, n=780 patients). AVANT, Bevacizumab-Avastin® adjuVANT trial. CV, cross-validation. HPC, histomorphological phenotype cluster. OS, overall survival. TCGA, The Cancer Genome Atlas. n, number of patients included in the analyses after excluding those with missing clinical data. Source data are provided as a Source Data file.

References

[1] Mesker, W. E. *et al.* The carcinoma–stromal ratio of colon carcinoma is an independent factor for survival compared to lymph node status and tumor stage. *Anal. Cell. Pathol.* **29**, 387–398 (2007).

[2] Friedl, P. & Alexander, S. Cancer invasion and the microenvironment: plasticity and reciprocity. *Cell* **147**, 992–1009 (2011).

[3] Zunder, S. M. *et al.* Predictive potential of tumour-stroma ratio on benefit from adjuvant bevacizumab in high-risk stage ii and stage iii colon cancer. *Br. J. Cancer* **119**, 164–169 (2018).

[4] Zunder, S. M., Gelderblom, H., Tollenaar, R. A. & Mesker, W. E. The significance of stromal collagen organization in cancer tissue: An in-depth discussion of literature. *Crit. Rev. Oncol.* **151**, 102907 (2020).

[5] Ueno, H. *et al.* Histopathological atlas of desmoplastic reaction characterization in colorectal cancer. *Jpn. J. Clin. Oncol.* **51**, 1004–1012 (2021).

[6] Strous, M. *et al.* A high tumour-stroma ratio (tsr) in colon tumours and its metastatic lymph nodes predicts poor cancer-free survival and chemo resistance. *Clin. Transl. Oncol.* **24**, 1047–1058 (2022).

[7] Polack, M. *et al.* Results from the united study: a multicenter study validating the prognostic effect of the tumor–stroma ratio in colon cancer. *ESMO open* **9**, 102988 (2024).

[8] Weiser, M. R., Gönen, M., Chou, J. F., Kattan, M. W. & Schrag, D. Predicting survival after curative colectomy for cancer: individualizing colon cancer staging. *J. Clin. Oncol.* **29**, 4796 (2011).

[9] Ueno, H. *et al.* New criteria for histologic grading of colorectal cancer. *The Am. journal surgical pathology* **36**, 193–201 (2012).

[10] Brierley, J. D., Gospodarowicz, M. K. & Wittekind, C. *TNM classification of malignant tumours* (John Wiley & Sons, 2017).

[11] Weiser, M. R. Ajcc 8th edition: colorectal cancer. *Ann. surgical oncology* **25**, 1454–1455 (2018).

[12] Pagès, F. *et al.* International validation of the consensus immunoscore for the classification of colon cancer: a prognostic and accuracy study. *The Lancet* **391**, 2128–2139 (2018).

[13] Bagaev, A. *et al.* Conserved pan-cancer microenvironment subtypes predict response to immunotherapy. *Cancer cell* **39**, 845–865 (2021).

[14] Ferrara, N. Vegf and the quest for tumour angiogenesis factors. *Nat. Rev. Cancer* **2**, 795–803 (2002).

[15] Hurwitz, H. *et al.* Bevacizumab plus irinotecan, fluorouracil, and leucovorin for metastatic colorectal cancer. *New Engl. journal medicine* **350**, 2335–2342 (2004).

[16] Kap, E. J. *et al.* Genetic variants in dna repair genes as potential predictive markers for oxaliplatin chemotherapy in colorectal cancer. *The pharmacogenomics journal* **15**, 505–512 (2015).

[17] Roos, W. P., Thomas, A. D. & Kaina, B. Dna damage and the balance between survival and death in cancer biology. *Nat. Rev. Cancer* **16**, 20–33 (2016).

[18] Vodicka, P., Vodenkova, S., Buchler, T. & Vodickova, L. Dna repair capacity and response to treatment of colon cancer. *Pharmacogenomics* **20**, 1225–1233 (2019).

[19] Ullman, T. A. & Itzkowitz, S. H. Intestinal inflammation and cancer. *Gastroenterology* **140**, 1807–1816 (2011).

[20] Lochhead, P. *et al.* Etiologic field effect: reappraisal of the field effect concept in cancer predisposition and progression. *Modern Pathol.* **28**, 14–29 (2015).

[21] Sandberg, T. P. *et al.* Increased expression of cancer-associated fibroblast markers at the invasive front and its association with tumor-stroma ratio in colorectal cancer. *BMC cancer* **19**, 1–9 (2019).

[22] Zins, K., Abraham, D., Sioud, M. & Aharinejad, S. Colon cancer cell–derived tumor necrosis factor-α mediates the tumor growth–promoting response in macrophages by up-regulating the colony-stimulating factor-1 pathway. *Cancer research* **67**, 1038–1045 (2007).

[23] Chang, S.-C. *et al.* Mutation spectra of common cancer-associated genes in different phenotypes of colorectal carcinoma without distant metastasis. *Ann. surgical oncology* **23**, 849–855 (2016).

[24] Shia, J. *et al.* Morphological characterization of colorectal cancers in the cancer genome atlas reveals distinct morphology–molecular associations: clinical and biological implications. *Modern pathology* **30**, 599–609 (2017).

[25] Nagtegaal, I., Arends, M. & Odze, R. Tumours of the colon and rectum: Who classification of tumours of the colon and rectum, tnm staging of carcinomas of the colon and rectum and the introduction. In *World Health Organization Classification of Tumours of the Digestive System*, 157–162 (IARC press, 2019).

[26] Zhu, G., Pei, L., Xia, H., Tang, Q. & Bi, F. Role of oncogenic kras in the prognosis, diagnosis and treatment of colorectal cancer. *Mol. cancer* **20**, 1–17 (2021).

[27] Jorgovanovic, D., Song, M., Wang, L. & Zhang, Y. Roles of ifn-γ in tumor progression and regression: a review. *Biomark. research* **8**, 1–16 (2020).

[28] Boland, C. R. & Goel, A. Microsatellite instability in colorectal cancer. *Gastroenterology* **138**, 2073–2087 (2010).

[29] Lugli, A. *et al.* Recommendations for reporting tumor budding in colorectal cancer based on the international tumor budding consensus conference (itbcc) 2016. *Modern pathology* **30**, 1299–1311 (2017).

[30] Brown, K. A. & Scherer, P. E. Update on adipose tissue and cancer. *Endocr. reviews* **44**, 961–974 (2023).
