# Supplementary material for: Self-supervised learning reveals clinically relevant histomorphological patterns for therapeutic strategies in colon cancer
Source: Nat Commun. 2025 Mar 8;16:2328. doi: 10.1038/s41467-025-57541-y (PMC11890774; doi:10.1038/s41467-025-57541-y)
Supplement: Supplementary file 2 — Reporting Summary [file 41467_2025_57541_MOESM2_ESM.pdf]

nature portfolio

Corresponding author(s): Prof. Aristotelis Tsirigos, Ph.D.
aristotelis.tsirigos@nyulangone.org

Last updated by author(s): Feb 6, 2025

Reporting Summary

Nature Portfolio wishes to improve the reproducibility of the work that we publish. This form provides structure for consistency and transparency in reporting. For further information on Nature Portfolio policies, see our Editorial Policies and the Editorial Policy Checklist.

Statistics

For all statistical analyses, confirm that the following items are present in the figure legend, table legend, main text, or Methods section.

n/a	Confirmed	
☐	☒	The exact sample size (n) for each experimental group/condition, given as a discrete number and unit of measurement
☐	☒	A statement on whether measurements were taken from distinct samples or whether the same sample was measured repeatedly
☐	☒	The statistical test(s) used AND whether they are one- or two-sided<br>*Only common tests should be described solely by name; describe more complex techniques in the Methods section.*
☐	☒	A description of all covariates tested
☐	☒	A description of any assumptions or corrections, such as tests of normality and adjustment for multiple comparisons
☐	☒	A full description of the statistical parameters including central tendency (e.g. means) or other basic estimates (e.g. regression coefficient) AND variation (e.g. standard deviation) or associated estimates of uncertainty (e.g. confidence intervals)
☐	☒	For null hypothesis testing, the test statistic (e.g. F, t, r) with confidence intervals, effect sizes, degrees of freedom and P value noted<br>*Give P values as exact values whenever suitable.*
☒	☐	For Bayesian analysis, information on the choice of priors and Markov chain Monte Carlo settings
☐	☒	For hierarchical and complex designs, identification of the appropriate level for tests and full reporting of outcomes
☐	☒	Estimates of effect sizes (e.g. Cohen's d, Pearson's r), indicating how they were calculated

Our web collection on statistics for biologists contains articles on many of the points above.

Software and code

Policy information about availability of computer code

Data collection	The training set included TCGA-COAD (i.e. The Cancer Genome Atlas Colon Adenocarcinoma) dataset consisting of 451 hematoxylin-and-eosin (H&E) whole slide images (WSIs) from 444 unique patients. We excluded duplications and WSIs with erroneous resolution that were not suitable for the analyses. The final TCGA-COAD training set included 435 WSIs from 428 patients with a diagnosed pathological TNM-stage I-IV colon carcinoma.<br><br>External test set was from a subset (N=1213) of the AVANT trial (phase III - Bevacizumab-Avastin® adjuVANT (BO17920)- Genentech Incl, Roche). The H&E WSIs were originally scanned at Leiden University Medical Center (LUMC) using a Panoramic 250 scanner (3DHistech, Hungary) at 20x magnification (tissue level pixel size ~0.33 µm/pixel).<br><br>For model interpretation, we linked the discovered histomorphological phenotype clusters (HPCs) to RNASeq-derived immune features (available for 131 patients) and gene expression of total 20,530 genes (available for 282 patients) using data within TCGA-COAD.
Data analysis	The code to replicate our results is available at: https://github.com/AdalbertoCq/Histomorphological-Phenotype-Learning (release 1)<br>The repository provides instructions to replicate results, any output file from the methodology, and Python version and software packages versions. The typical time it takes to train the Barlow-Twins on a single GPU station can take 2-3 days.

For manuscripts utilizing custom algorithms or software that are central to the research but not yet described in published literature, software must be made available to editors and reviewers. We strongly encourage code deposition in a community repository (e.g. GitHub). See the Nature Portfolio guidelines for submitting code & software for further information.

Data

Policy information about availability of data

All manuscripts must include a data availability statement. This statement should provide the following information, where applicable:

- Accession codes, unique identifiers, or web links for publicly available datasets
- A description of any restrictions on data availability
- For clinical datasets or third party data, please ensure that the statement adheres to our policy

The publicly available TCGA-COAD can be accessed at Genomic Data Commons portal (https://gdc.cancer.gov/). The immune landscape signatures for TCGA-COAD is available in Thorsson et al (2018). Accession ID is TCGA-COAD (colon adenocarcinoma), and https://www.cancer.gov/ccg/research/genomesequencing/tcga accessed on 2023-05-09 for additional immune landscape and gene set enrichment analyses. The AVANT data (BO17920) that support the findings of this study are available from Genentech Inc., Roche. However, access to these data is restricted as they were used under license for the current study and are not for commercial use to protect patient privacy. Data may however be available from the authors upon request for research purposes with permission from Genentech Inc., Roche. Data analyses are based upon publicly available Python software packages and codes are available from our previous publication (Quiros et al [2024]) (https://github.com/AdalbertoCq/Histomorphological-Phenotype-Learning). The remaining data are available within the Article, Supplementary Information or Source Data file.

Research involving human participants, their data, or biological material

Policy information about studies with human participants or human data. See also policy information about sex, gender (identity/presentation), and sexual orientation and race, ethnicity and racism.

Reporting on sex and gender	This study was trained on datasets which include both male and female participants. Sex- and gender-based analyses were not performed in this study.
Reporting on race, ethnicity, or other socially relevant groupings	Race, ethnicity, or other socially relevant groupings information were not considered in the study.
Population characteristics	Detailed population characteristics were not described or analyzed in this study. However, the characteristics of the AVANT external test set used in the current study correspond to:<br>"Stéphanie M Zunder, Gabi W van Pelt, Hans J Gelderblom, Christoph Mancao, Hein Putter, Rob A Tollenaar, and Wilma E Mesker. Predictive potential of tumour-stroma ratio on benefit from adjuvant bevacizumab in high-risk stage ii and stage iii colon cancer. British Journal of Cancer, 119(2):164–169, 2018." Population characteristics for TCGA-COAD can be retrieved at https://portal.gdc.cancer.gov/
Recruitment	The recruitment protocols of TCGA participants can be found on the websites: https://portal.gdc.cancer.gov/<br><br>The recruitment of the AVANT trial (BO17920) can be found on https://classic.clinicaltrials.gov/ct2/show/NCT00112918 or in their published article:<br>"Aimery de Gramont, Eric Van Cutsem, Hans-Joachim Schmoll, Josep Tabernero, Stephen Clarke, Malcolm J Moore, David Cunningham, Thomas H Cartwright, J Randolph Hecht, Fernando Rivera, et al. Bevacizumab plus oxaliplatin-based chemotherapy as adjuvant treatment for colon cancer (AVANT) a phase 3 randomised controlled trial. The lancet oncology, 13(12):1225–1233, 2012."
Ethics oversight	The present study was performed by using anonymized archival material, not necessitating new informed consent. Archival material was derived from the AVANT-trial (BO17920), performed in accordance with the declaration of Helsinki, as seen in de Gramont et al (2012). TCGA-COAD dataset is publicly available and no further informed consent was required.

Note that full information on the approval of the study protocol must also be provided in the manuscript.

Field-specific reporting

Please select the one below that is the best fit for your research. If you are not sure, read the appropriate sections before making your selection.

☒ Life sciences ☐ Behavioural & social sciences ☐ Ecological, evolutionary & environmental sciences

For a reference copy of the document with all sections, see nature.com/documents/nr-reporting-summary-flat.pdf

Life sciences study design

All studies must disclose on these points even when the disclosure is negative.

Sample size	The TCGA-COAD dataset consisted of 451 H&E surgical WSIs from 444 unique patients, with a subset of patients were also available on matched genetic and transcriptomic information. After exclusion of duplications and WSIs with erroneous resolution, the final sample included for training was 435 WSIs from 428 patients with a diagnosed pathological TNM-stage I-IV colon carcinoma. The external test set was derived from a subset within the AVANT trial, as described by Zunder et al (2018). The external test set included 1,213 patients, each with an available H&E WSI from diagnostic surgical specimens.
Data exclusions	We excluded duplications and WSIs with erroneous resolution that were not suitable for the analyses (i.e. only several kilobytes in size) from

	the TCGA-COAD. Original exclusion criteria for AVANT trial was published in Zunder et al (2018), no additional exclusion criteria were applied to our external test set.
Replication	We used 5-fold cross-validation within the training set and external validation in one independent test set.
Randomization	A 5-fold cross-validation split was randomly generated, stratified by patient clinical variables within TCGA.
Blinding	During the pathological assessment of the discovered HPCs, the assessors were blinded with the inter-connections among HPCs as well as any patient outcomes and results from any subsequent analyses. During the objective test for morphological consistency within each HPC, the assessor was blinded for the HPC labels, as well as patient outcomes and results from any subsequent analyses.

Reporting for specific materials, systems and methods

We require information from authors about some types of materials, experimental systems and methods used in many studies. Here, indicate whether each material, system or method listed is relevant to your study. If you are not sure if a list item applies to your research, read the appropriate section before selecting a response.

Materials & experimental systems

n/a	Involved in the study
☒	☐ Antibodies
☒	☐ Eukaryotic cell lines
☒	☐ Palaeontology and archaeology
☒	☐ Animals and other organisms
☐	☒ Clinical data
☒	☐ Dual use research of concern
☒	☐ Plants

Methods

n/a	Involved in the study
☒	☐ ChIP-seq
☒	☐ Flow cytometry
☒	☐ MRI-based neuroimaging

Clinical data

Policy information about clinical studies

All manuscripts should comply with the ICMJE guidelines for publication of clinical research and a completed CONSORT checklist must be included with all submissions.

Clinical trial registration	The AVANT trial (BO17920, Genentech Inc. Roche) can be found on https://classic.clinicaltrials.gov/ct2/show/NCT00112918 or in their published article:<br>"Aimery de Gramont, Eric Van Cutsem, Hans-Joachim Schmoll, Josep Tabernero, Stephen Clarke, Malcolm J Moore, David Cunningham, Thomas H Cartwright, J Randolph Hecht, Fernando Rivera, et al. Bevacizumab plus oxaliplatin-based chemotherapy as adjuvant treatment for colon cancer (AVANT) a phase 3 randomised controlled trial. The Lancet Oncology, 13(12):1225–1233, 2012."
Study protocol	The study protocol from the AVANT trial can be found on https://classic.clinicaltrials.gov/ct2/show/NCT00112918
Data collection	Data collection as according to protocol can be found on https://classic.clinicaltrials.gov/ct2/show/NCT00112918 or for the results of collection, in the published article from de Gramont et al (2012). Premature termination of the trial due to adverse outcomes was initiated.
Outcomes	Results from the complete phase III AVANT trial can be found on https://classic.clinicaltrials.gov/ct2/show/results/NCT00112918 or in their published article from de Gramont et al (2012). In conclusion; Bevacizumab does not prolong disease-free survival when added to adjuvant chemotherapy in resected stage III colon cancer. Overall survival data suggest a potential detrimental effect with bevacizumab plus oxaliplatin-based adjuvant therapy in these patients.

Plants

Seed stocks	*Report on the source of all seed stocks or other plant material used. If applicable, state the seed stock centre and catalogue number. If plant specimens were collected from the field, describe the collection location, date and sampling procedures.*
Novel plant genotypes	*Describe the methods by which all novel plant genotypes were produced. This includes those generated by transgenic approaches, gene editing, chemical/radiation-based mutagenesis and hybridization. For transgenic lines, describe the transformation method, the number of independent lines analyzed and the generation upon which experiments were performed. For gene-edited lines, describe the editor used, the endogenous sequence targeted for editing, the targeting guide RNA sequence (if applicable) and how the editor was applied.*
Authentication	*Describe any authentication procedures for each seed stock used or novel genotype generated. Describe any experiments used to assess the effect of a mutation and, where applicable, how potential secondary effects (e.g. second site T-DNA insertions, mosiacism, off-target gene editing) were examined.*
